# Optic Neuritis in Temporal Association With Cosmetic Hyaluronic Acid Injection: A Case Requiring Differentiation From Ischemic Optic Neuropathy

**DOI:** 10.7759/cureus.109226

**Published:** 2026-05-19

**Authors:** Ryo Ogasawara, Yasuyuki Takai, Akiko Yamagami, Kenji Inoue

**Affiliations:** 1 Ophthalmology, Inouye Eye Hospital, Tokyo, JPN

**Keywords:** gadolinium-enhanced mri, hyaluronic acid, intravenous immunoglobulin, ischemic optic neuropathy, optic neuritis, optic neuropathy

## Abstract

Visual impairment following cosmetic facial hyaluronic acid injection is frequently attributed to ischemic etiologies, but non-ischemic causes can make the diagnosis challenging. We report a case of optic neuritis that developed after hyaluronic acid injection.

A 27-year-old woman developed painless visual loss and a visual field defect in the left eye several days after cosmetic hyaluronic acid injection in both cheeks. The symptoms progressed over approximately two weeks, and she was referred to our hospital. Visual acuity was 1.2 in the right eye and 0.15 in the left eye. A left relative afferent pupillary defect was present. Goldmann perimetry (GP) demonstrated a central scotoma and peripheral visual field constriction in the left eye. Fundus examination showed no abnormalities. Contrast-enhanced magnetic resonance imaging (MRI) of the left optic nerve showed hyperintensity on short tau inversion recovery (STIR) and enhancement on gadolinium (Gd)-enhanced fat-suppressed T1-weighted images. Blood tests showed no systemic inflammation, and both anti-aquaporin-4 (AQP4) and anti-myelin oligodendrocyte glycoprotein (MOG) antibodies were negative. The patient was diagnosed with left optic neuritis. She received three courses of intravenous methylprednisolone pulse therapy, followed by intravenous immunoglobulin therapy. Best-corrected visual acuity in the left eye improved to 0.7 at two months and 0.9 at four months after onset.

In this case, a direct relationship between hyaluronic acid injection and optic neuropathy could not be established. However, when visual loss develops after cosmetic procedures, the differential diagnosis should include not only the commonly reported ischemic disorders but also inflammatory optic neuropathy.

## Introduction

The number of cosmetic procedures has increased in recent years, particularly nonsurgical treatments using injectable materials such as hyaluronic acid. Facial hyaluronic acid injection is widely performed for aesthetic purposes. In addition to common adverse effects, such as allergic reactions and bruising, rare but severe ocular ischemic complications - including retinal artery occlusion and ischemic optic neuropathy - have been reported, sometimes resulting in permanent vision loss [1]. Accordingly, ischemic ocular disorders are usually the first diagnostic consideration when visual dysfunction develops after hyaluronic acid injection. Ischemic optic neuropathy is caused by impaired blood supply to the optic nerve, whereas optic neuritis is an inflammatory optic neuropathy. These conditions may share overlapping clinical features and can sometimes be difficult to differentiate clinically, particularly in the acute phase.

An association between hyaluronic acid injection and inflammatory ocular disease, including optic neuritis and optic perineuritis, has not been established. However, when inflammatory or immune-mediated non-ischemic etiologies are considered, the diagnosis can be challenging, and careful differentiation from ischemic ocular disorders is essential. We report a case of optic neuritis following cosmetic hyaluronic acid injection and highlight the clinical and imaging features that help distinguish it from ischemic optic neuropathy. This is a single case report; therefore, institutional review board approval was not required. Written informed consent was obtained from the patient for publication of this case report and accompanying images.

## Case presentation

A 27-year-old woman received hyaluronic acid injections in both cheeks for cosmetic purposes. Several days after the procedure, painless visual loss and visual field constriction gradually developed in the left eye, while no visual symptoms developed in the right eye. The patient denied headache, tinnitus, vomiting, transient visual obscurations, diplopia, or other systemic symptoms. Two weeks later, she noticed blurred vision and a visual field defect involving approximately one quarter of the inferotemporal field in the left eye. Because the visual loss and visual field defect continued to progress, she was referred to our hospital.

At the initial visit, visual acuity was 1.2 without correction in the right eye and 0.15 (corrected with +0.50 DS/-0.75 DC × 165°) in the left eye. Intraocular pressure was 13.0 mmHg in the right eye and 14.0 mmHg in the left eye. Critical flicker frequency was 43 Hz in the right eye and 17 Hz in the left eye. A left relative afferent pupillary defect (RAPD) was present. Goldmann perimetry (GP) showed a central scotoma and marked peripheral visual field constriction in the inferotemporal field of the left eye, while no significant visual field abnormality was observed in the right eye (Figure 1A, 1B). No abnormalities were found in the anterior segment, lens, or vitreous. The fundus showed no retinal discoloration, and the optic disc was normal (Figure 1C, 1D). Fluorescein angiography (FA) demonstrated no leakage from the optic disc and no evidence of ischemia, with no filling delay of the optic disc or peripapillary choroid (Figure 1E, 1F). Optical coherence tomography (OCT) revealed no abnormalities in the macula or the peripapillary retinal nerve fiber layer (Figure 2A-2E).

**Figure 1:**
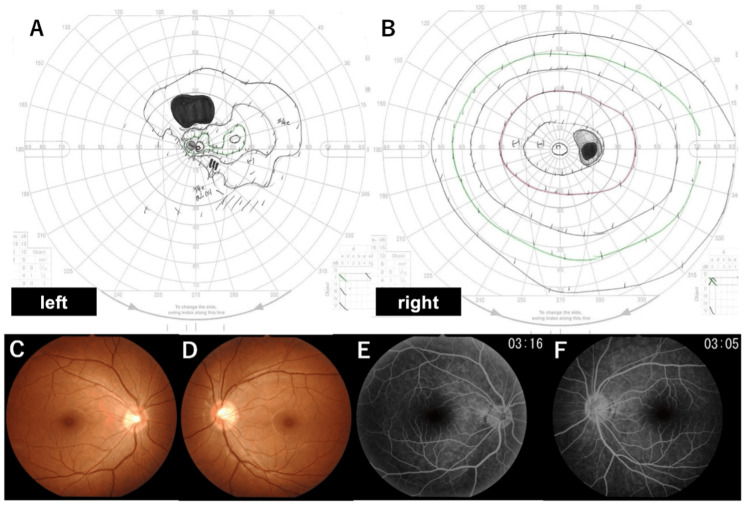
Goldmann perimetry, fundus images, and fluorescein angiography findings at the initial visit A (left eye, GP): A central scotoma and concentric peripheral visual field constriction are present. B (right eye, GP): No abnormalities are observed. C (left fundus), D (right fundus): The optic disc color is preserved bilaterally, with no vascular abnormalities. E (left eye, FA), F (right eye, FA): There is no leakage from the optic disc, no evidence of ischemia, and no filling delay of the optic disc or peripapillary choroid. GP: Goldmann perimetry; FA: fluorescein angiography

**Figure 2:**
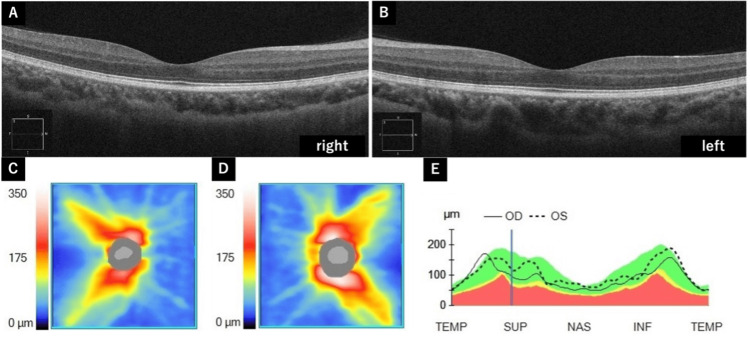
Optical coherence tomography findings at the initial visit A (right eye, macular B-scan), B (left eye, macular B-scan): No abnormalities are observed in the macula of either eye. C (right eye, peripapillary retinal nerve fiber layer (RNFL) thickness map), D (left eye, RNFL thickness map): The peripapillary RNFL thickness is within normal limits bilaterally. E (peripapillary RNFL thickness profile): No swelling or thinning is observed in either eye (OD, solid line; OS, dashed line).

Although fundus findings were normal, reduced visual acuity, visual field defect, and positive RAPD were suggestive of left optic neuropathy. Because visual dysfunction developed soon after cosmetic hyaluronic acid injection, ischemic optic neuropathy due to vascular occlusion was initially considered. Therefore, contrast-enhanced magnetic resonance imaging (MRI), including diffusion-weighted imaging (DWI), was performed to distinguish inflammatory optic neuropathy from ischemic or compressive optic neuropathy. Contrast-enhanced MRI showed hyperintensity on short tau inversion recovery (STIR) sequences along the left optic nerve, extending from the posterior orbital segment to the intracanalicular segment (Figure 3A, 3B) and enhancement on gadolinium (Gd)-enhanced fat-suppressed T1-weighted images (Figure 3C, 3D). No abnormalities were seen on DWI or the apparent diffusion coefficient (ADC) map (Figure 3E, 3F). Blood tests showed no systemic inflammation. Both anti-aquaporin-4 (AQP4) and anti-myelin oligodendrocyte glycoprotein (MOG) antibodies were negative, and no other notable abnormalities were identified.

**Figure 3:**
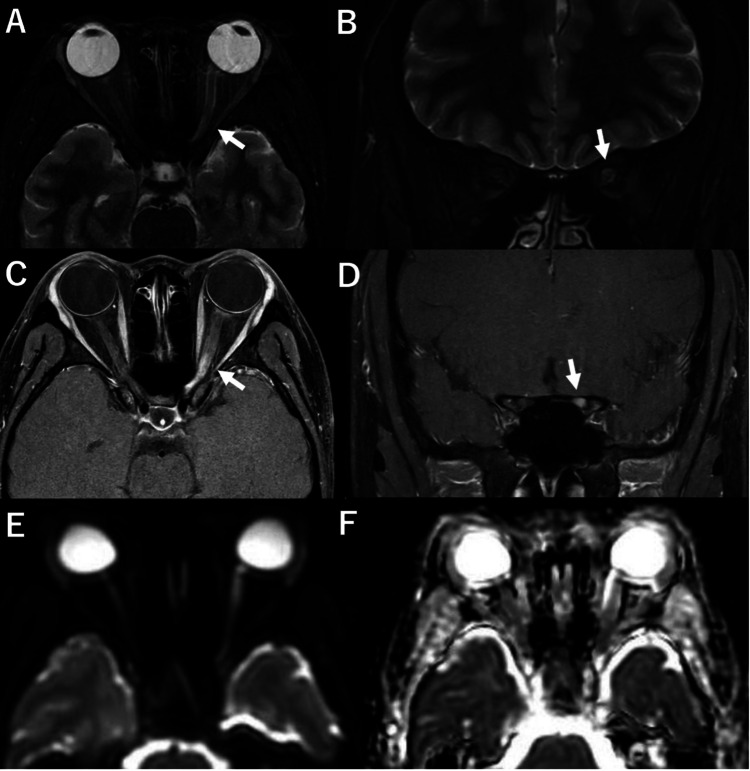
Magnetic resonance imaging findings at the initial visit A (short tau inversion recovery (STIR), axial view), B (STIR, coronal view): Hyperintensity is present in the left optic nerve, extending from the posterior orbital segment to the intracanalicular segment. C (gadolinium (Gd)-enhanced fat-suppressed T1-weighted image, axial view), D (Gd-enhanced fat-suppressed T1-weighted image, coronal view): Contrast enhancement is evident along the left optic nerve. E (diffusion-weighted imaging (DWI), axial view), F (apparent diffusion coefficient (ADC) map, axial view): No diffusion restriction is observed in the optic nerve, and there are no findings suggestive of ischemia.

Because symptoms developed soon after the cosmetic procedure, posterior ischemic optic neuropathy (PION) was initially considered. However, findings suggestive of ischemia were lacking, and MRI demonstrated contrast-enhancing optic nerve abnormalities; she was therefore diagnosed with left optic neuritis. She received three courses of intravenous methylprednisolone pulse therapy (1,000 mg/day for three days per course). Because the central scotoma and visual field impairment persisted despite corticosteroid therapy, intravenous immunoglobulin therapy (400 mg/kg/day for five days) was administered as additional immunotherapy. Best-corrected visual acuity in the left eye improved to 0.7 at two months and to 0.9 at four months after onset. Follow-up MRI showed a reduction of the contrast enhancement in the left optic nerve (Figure 4A, 4B), and GP showed a smaller central scotoma and expansion of the peripheral visual field (Figure 4C, 4D). Oral prednisolone was started at 20 mg/day, gradually tapered, and discontinued after one year. At the final visit, visual acuity in the left eye was 1.0, with no recurrence.

**Figure 4:**
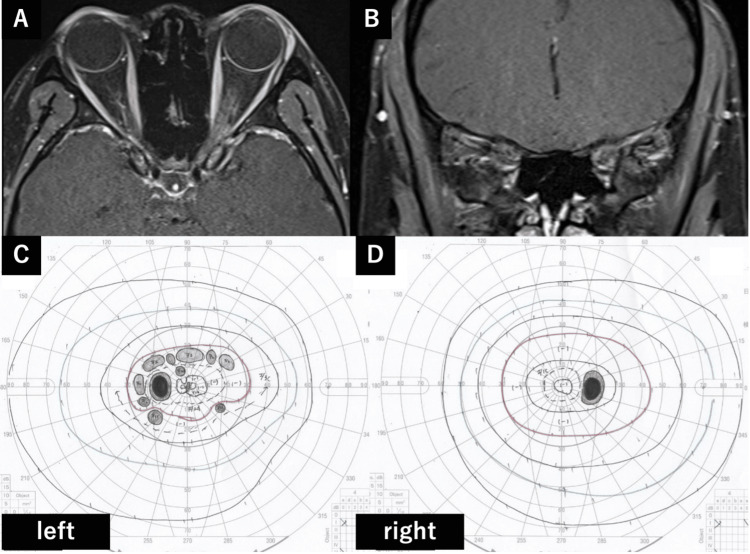
Follow-up magnetic resonance imaging and Goldmann perimetry findings after treatment A (gadolinium (Gd)-enhanced fat-suppressed T1-weighted image, axial view), B (Gd-enhanced fat-suppressed T1-weighted image, coronal view): After two courses of intravenous methylprednisolone pulse therapy, contrast enhancement of the left optic nerve has decreased compared with the initial examination. C (left eye, GP): After completion of intravenous methylprednisolone pulse therapy and intravenous immunoglobulin therapy, the central scotoma markedly decreased and the peripheral visual field expanded. D (right eye, GP): No abnormalities are observed. GP: Goldmann perimetry

## Discussion

This patient developed visual dysfunction after facial hyaluronic acid injection and presented with left-sided optic neuropathy. The temporal relationship between symptom onset and the cosmetic procedure prompted consideration of a possible association with hyaluronic acid injection rather than idiopathic optic neuritis alone. Although fundus examination was unremarkable and both AQP4 and MOG antibodies were negative, contrast-enhanced MRI demonstrated a lesion in the left optic nerve, and the patient responded to corticosteroid and immunoglobulin therapy. Overall, the clinical course was consistent with optic neuritis. However, a direct causal relationship between hyaluronic acid injection and optic neuritis could not be established. Because embolic ischemia is an important differential diagnosis after cosmetic procedures, PION also had to be considered.

Delayed inflammatory reactions and immune-mediated adverse events, including autoimmune/inflammatory syndrome induced by adjuvants (ASIA)-like phenomena, have been reported after hyaluronic acid filler injection [2,3], and these observations lend some support to an inflammatory mechanism. Previously reported delayed reactions, such as nodules and granulomatous changes, have been attributed to infection or biofilm formation, injection technique, and product characteristics [2]. However, these have mainly involved cutaneous and soft tissue reactions, and their relevance to optic neuritis is limited. Optic perineuritis after hyaluronic acid injection has been described [4], although the injection sites and clinical features vary. The present case, therefore, demonstrates optic neuritis in temporal association with hyaluronic acid injection without establishing direct causation. Rather than proving causation, this case highlights the broader differential diagnosis that should be considered in clinical practice. Unlike previously reported ischemic complications associated with embolic vascular occlusion, no evidence of embolism or ischemia was identified in the present case. Although the mechanism remains uncertain, a possible immune-mediated inflammatory reaction triggered by hyaluronic acid injection may be considered. However, coincidental optic neuritis unrelated to the cosmetic procedure cannot be excluded.

Most reported cases of visual loss after facial hyaluronic acid filler injection have involved embolic ischemic events, including retinal artery occlusion, ophthalmic artery occlusion, and cerebral infarction [5-9]. Anatomically, injection sites, such as the glabella, nose, and cheek, are connected to branches of the ophthalmic artery or their anastomoses. When filler material enters a vessel, it can reach the ophthalmic arterial system via retrograde flow, which is thought to underlie these ischemic ocular complications [5-7]. Previous reviews have shown that severe vision loss most often results from occlusion within the ophthalmic arterial system, whereas PION is relatively uncommon [7]. Nevertheless, ischemic optic neuropathy, including PION, should remain in the differential diagnosis of acute vision loss after cosmetic procedures, even when fundus findings are unremarkable. Because cerebral infarction and ischemia of the orbital adnexa may coexist, systemic imaging should complement the ophthalmic examination [8,9]. Compared with previously reported embolic ischemic complications after cosmetic filler injection, the present case showed predominantly inflammatory imaging findings without evidence of vascular occlusion.

PION is a rare subtype of ischemic optic neuropathy that involves the retrobulbar portion of the optic nerve and, unlike anterior ischemic optic neuropathy, typically presents with acute painless vision loss without optic disc swelling [10]. PION has been reported in association with perioperative hypotension and major hemorrhage [11], as well as with giant cell arteritis [10,12]. Because clinical differentiation from optic neuritis can be difficult [13], MRI plays a central role in distinguishing the two conditions. PION may show ischemic changes such as high signal on DWI with corresponding low signal on ADC [14,15]. In the present case, however, DWI and ADC showed no findings suggestive of ischemia, whereas STIR hyperintensity and contrast enhancement were seen in the optic nerve, and the patient responded to immunotherapy. Taken together, these findings were considered less consistent with a typical embolic ischemic pattern and more suggestive of inflammatory optic neuropathy, although complete differentiation from atypical inflammatory PION may not be possible. Other differential diagnoses, including compressive optic neuropathy, demyelinating disease, and systemic inflammatory disorders, were also considered. However, the absence of compressive lesions on MRI, negative anti-AQP4 and anti-MOG antibodies, lack of systemic inflammatory findings, and favorable response to immunotherapy supported the diagnosis of inflammatory optic neuropathy.

## Conclusions

Although a direct causal relationship between hyaluronic acid injection and optic neuritis could not be established, the temporal association in this case raises the possibility that cosmetic hyaluronic acid injection may have acted as a trigger for inflammatory optic neuropathy. However, the possibility of coincidental optic neuritis unrelated to the cosmetic procedure cannot be excluded. The differential diagnosis of visual loss after cosmetic procedures should therefore include inflammatory optic neuropathy in addition to the more commonly reported embolic ischemic complications. Contrast-enhanced MRI, combined with neuro-ophthalmic assessment, is essential for distinguishing ischemic and inflammatory etiologies and for guiding prompt, appropriate treatment. Early recognition of this rare presentation may help clinicians avoid delays in initiating immunotherapy, which, in our case, was associated with substantial visual recovery.
